# 187. Vancomycin Plus Ceftaroline Salvage Therapy for Persistent Methicillin-Resistant *Staphylococcus aureus* Bacteremia

**DOI:** 10.1093/ofid/ofab466.389

**Published:** 2021-12-04

**Authors:** Wesley D Kufel, Katie A Parsels, Jeffrey Steele, Robert Seabury, Kristopher M Paolino, Stephen J Thomas

**Affiliations:** 1 Binghamton University School of Pharmacy and Pharmaceutical Sciences, Binghamton, New York; 2 Upstate University Hospital, Syracuse, New York; 3 State University of New York Upstate University Hospital, Syracuse, NY; 4 SUNY Upstate University Hospital, Syracuse, NY; 5 State University of New York Upstate Medical University, Syracuse, New York

## Abstract

**Background:**

The preferred antibiotic salvage regimen for persistent methicillin-resistant *Staphylococcus aureus* bacteremia (pMRSAB) is unclear. Vancomycin plus ceftaroline (V/C) has demonstrated potent *in vitro* synergistic activity against MRSA; however, clinical data is limited. Thus, we sought to evaluate V/C salvage therapy for pMRSAB.

**Methods:**

This was a single-center, retrospective cohort study of patients with MRSAB who received V/C salvage therapy between 1/1/2016-3/10/2021. Adult patients were included if blood cultures (BC) were positive for MRSA for ≥ 72 hours, received anti-MRSA monotherapy initially, and subsequently received V/C ≥ 24 hours. Patients were excluded if they received other anti-MRSA antibiotics within 72 hours of V/C initiation. The primary outcome was time to BC clearance following V/C initiation. Secondary outcomes included 90-day all-cause mortality, microbiological cure, 90-day MRSAB recurrence, and length of stay (LOS). Microbiological cure was defined as BC clearance.

**Results:**

Of 178 patients identified, 20 were evaluated after inclusion and exclusion criteria were applied. Mean (SD) age and Pitt Bacteremia score were 38.5 (14.5) years and 4.2 (3.1), respectively. Most patients were male (70%), intravenous drug users (65%), and admitted to the intensive care unit (65%). The most common source was intravenous drug use (55%) and the majority had infective endocarditis (70%). All patients received infectious disease consultation and median (IQR) vancomycin AUC:MIC was 527 (454, 611). Source control, if possible, was obtained in most patients (55%). Median (IQR) time to bloodstream clearance from first positive BC and from when ceftaroline was initiated was 9.7 (8.4, 10.2) and 2.4 (1.5, 3.1) days, respectively. 90-day all-cause mortality, microbiological cure, and 90-day MRSAB recurrence occurred in 35%, 95%, and 5% of patients, respectively. Median (IQR) LOS was 25 (14.5, 32.0) days.

**Conclusion:**

To our knowledge, this is the largest cohort to evaluate V/C for pMRSAB. Patients were medically complex; however, median time to MRSAB clearance following ceftaroline initiation was < 2.5 days and microbiological cure was obtained in nearly all patients. V/C may represent a potential salvage regimen for pMRSAB.

**Disclosures:**

**Wesley D. Kufel, PharmD**, **Melinta** (Research Grant or Support)**Merck** (Research Grant or Support)**Theratechnologies, Inc.** (Advisor or Review Panel member) **Jeffrey Steele, Pharm.D.**, **Paratek Pharmaceuticals** (Advisor or Review Panel member)

